# Psoriasis in the Context of Dermatologic Disorders: A Comprehensive Overview

**DOI:** 10.3390/diseases13100322

**Published:** 2025-10-01

**Authors:** Julia Nowowiejska-Purpurowicz, Patrycja Lemiesz, Iwona Flisiak

**Affiliations:** Department of Dermatology and Venereology, Medical University of Bialystok, 14 Zurawia St, 15-540 Bialystok, Poland; patrycjadrozd89@gmail.com (P.L.); iwona.flisiak@umb.edu.pl (I.F.)

**Keywords:** psoriasis, pustular psoriasis, ILVEN, atopic dermatitis, pityriasis rubra pilaris, Sneddon–Wilkinson disease, anti-p200 pemphigoid, seborrheic dermatitis

## Abstract

Psoriasis is a chronic, immune-mediated dermatosis that affects approximately 125 million people worldwide. Traditionally considered a dermatologic condition, it is now perceived as a systemic disease with numerous comorbidities. While its associations with psoriatic arthritis, metabolic syndrome, and psychiatric disorders are well established, less attention has been given to its coexistence with other dermatoses. This narrative review aims to explore and summarize the existing evidence on the relationships between psoriasis and other skin diseases, highlighting potential overlaps in clinical presentation, pathogenesis, and treatment challenges. Psoriasis may coexist with several inflammatory and autoimmune skin disorders, including atopic dermatitis, lichen simplex chronicus, anti-p200 pemphigoid, pityriasis rubra pilaris, seborrheic dermatitis, inflammatory linear verrucous nevus (ILVEN), Sneddon–Wilkinson disease, and vitiligo. The review highlights the shared genetic pathways (e.g., the Th1/Th17 axis and IL-17 pathway), diagnostic challenges (e.g., sebopsoriasis and psoriasis–eczema overlap), and therapeutic considerations (e.g., paradoxical reactions to biologics and effectiveness of JAK inhibitors in both psoriasis and vitiligo). The coexistence of psoriasis with other dermatoses is more common and clinically significant than previously appreciated. Recognizing these associations is crucial for an accurate diagnosis, avoiding mismanagement, and optimizing individualized treatment strategies. Further research is needed to elucidate the underlying mechanisms and improve the multidisciplinary care for patients with complex dermatologic presentations.

## 1. Introduction

Psoriasis has been a mainstay of dermatology for many years. It is one of the most common diseases in the daily medical practice of most dermatologists, as well as the subject of much scientific research. Interestingly, despite so many studies, it is still not fully understood; the autoimmune pathway has still not been fully elucidated, and new molecules that may participate in its pathogenesis are continuously being discovered [1].

Psoriasis affects 125 million people worldwide [1] and is therefore a significant medical and economic problem. Its prevalence varies in different geographical regions; for instance, it is more common in Scandinavia and less common in Asia [1]. Men and women seem to be equally affected, but adults are more often affected than children [1]. Continually deepening our knowledge on it should be a priority in the field of dermatology. The clinical presentation of the most frequent type of psoriasis—plaques—is well known; it involves erythematous, scaly plaques located on the extensor surfaces of elbows and knees, as well as in the lumbosacral area, intragluteal fold, or scalp. Nevertheless, in severe cases, the whole body may be affected [1].

Nowadays, there is a lot of talk about the comorbidity of psoriasis, which means that it may be related to other independent diseases. It is now being treated as a kind of systemic disease rather than one that only affects the skin. We already know a lot about the associations between psoriasis and other diseases, especially arthritis, metabolic syndrome, and psychiatric disorders [2], but not enough is known about its links to other skin diseases. The coexistence of several conditions with psoriasis has been well documented and may even be regarded as a spectrum of the same dermatosis. There are also case reports of somewhat less confirmed dermatoses that have been observed in psoriatic patients. However, there is no review focused on this topic.

The review aimed to study and summarize the association between psoriasis and other skin diseases and to contribute to the limited data in light of the paucity of available publications.

## 2. Psoriasis and Atopic Dermatitis and Lichen Simplex Chronicus

Atopic dermatitis (AD) is an inflammatory dermatosis in which, similar to psoriasis, immunological abnormalities occur in genetically predisposed individuals and after exposure to environmental stimuli. The essence of AD is a disruption of the epidermal barrier, resulting in water loss (clinically manifesting as dry skin) and greater penetration of allergens and pathogens, resulting in a higher incidence of allergies and secondary superinfections in this group of patients [3].

It has been found that certain filaggrin mutations may also increase the risk of developing psoriasis; this is true for Chinese and Taiwanese populations. In addition, IL-13 associated with chromosomal region 5q31.1-q33.1 plays a role in both psoriasis and AD. In terms of immune disorders, the similarity between psoriasis and AD relates to the involvement of Th1 and Th22 cells [4].

The clinical picture of AD varies depending on the age of the patient. In infants, the lesions are erythematous and papular in nature with the presence of vesicles and exudate, and are localized on the face and on the upright surfaces of the extremities. On the other hand, in adults, the lesions are erythematous and exfoliative and localized on the volar surfaces of the limbs. The skin lesions are accompanied by pruritus, which also results in the presence of excoriations [5]

Clinically, the diagnosis of psoriasis or AD, although usually straightforward, sometimes poses difficulties. Several clinical variants have even been proposed by Tsai et al. for the overlap between psoriasis and AD: (1) psoriasis with features of AD, (2) AD with features of psoriasis, (3) AD and psoriasis co-occurring, (4) development of lesions with AD morphology during treatment of psoriasis, and (5) development of psoriasis during treatment of AD [4].

**Figure 1:**
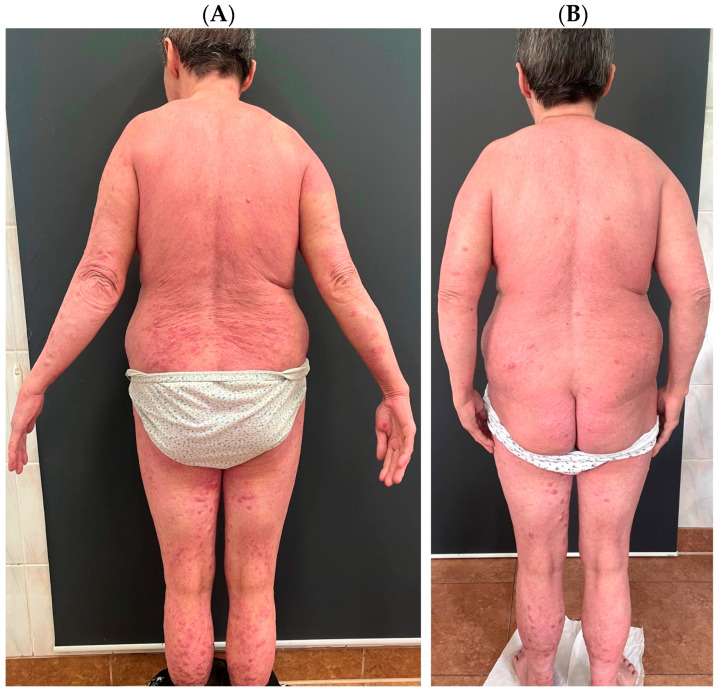
A patient who suffers from atopic dermatitis and psoriasis. The patient was diagnosed with psoriasis in childhood and treated topically; after puberty, atopic dermatitis was also diagnosed. The patient was treated in the past with ciclosporin A, methotrexate, acitretin, prednisone, UVB phototherapy, and PUVA. The patient was successfully treated with upadacitinib, which was chosen to cover both dermatoses simultaneously. (**A**) shows the patient before the introduction of upadacitinib; (**B**) shows the patient’s skin condition after one month of treatment.

Variants of psoriasis with features of AD (1) are primarily nummular psoriasis and psoriatic erythroderma. In the former, patients usually report severe pruritus, while in the latter, elevated IgE class antibodies and eosinophilia are observed. The opposite situation occurs when patients with AD present some clinical features of psoriasis (2). Such lesion morphologies are observed in the Asian phenotype. Clinical cases of co-occurrence of psoriasis with AD have been named “psoriasis dermatitis,” “eczema in psoriatico,” “PsEma,” or “PSO-Eczema” (3), and the incidence of such overlapping entities is estimated to be 0.2–16.7%, with a lower frequency in children. In these cases, the role of IL-17 in the pathogenesis is emphasized [4]. Figure 1 presents a patient who suffers from both AD and psoriasis who was successfully treated with upadacitinib.

It should be remembered that during the treatment of psoriasis, lesions with an eczematous morphology can develop (4). This sometimes occurs during biological treatments, especially with TNFα inhibitors, although cases have been described after using other types of drugs as well. Eczematous lesions can be limited or generalized with a typical AD morphology. A postulated explanation for this phenomenon is the inhibition of TNFα and the subsequent increased secretion of IFNα, leading to the appearance of lesions. Another explanation is genetic predisposition or IL-17 inhibition, which results in polarization of Th2 lymphocytes and increased inflammation [4].

The reverse is also possible (5). When AD is treated with dupilumab, a biological drug that blocks the receptor for IL-4 and inhibits transmission through IL-13, IL-4 activity is inhibited and it cannot inhibit Th17-dependent and IL-17-dependent responses, which are particularly important in the pathogenesis of psoriasis, and can result in the eruption of psoriatic lesions [6]. Table 1 summarizes the variants of psoriasis and AD overlap (Table 1).

The comorbidity of AD is also receiving increasing attention. It turns out that many of the disorders more frequently observed among patients with psoriasis also affect patients with AD. For example, both psoriasis and AD show a significant association with metabolic syndrome. In addition, patients with both dermatoses have a higher risk of developing alopecia areata, vitiligo, inflammatory bowel disease, or asthma [4].

Lichen simplex chronicus (LSC) is a chronic and mild skin disorder characterized by well-defined dry, scaly, and rough patches. It affects approximately 12% of the general population, and more frequently affects women than men. Skin lesions appear due to chronic scratching or rubbing [7]. LSC may occur as a primary disorder related to psychological factors since emotional disorders may lead to the compulsion of chronic scratching. On the other hand, LSC may also be secondary to other skin diseases associated with pruritus, such as atopic dermatitis, eczema, or psoriasis [7]. The treatment consists of topical glucocorticoids, moisturizers, and even occlusive procedures to cover the affected area. Oral antihistamines can be used to alleviate pruritus [7].

Besides some similarities in the clinical manifestations and the fact that psoriasis may lead to LSC, there is also a resemblance between LSC and psoriasis on the microscopic level since both are characterized by acanthosis and parakeratosis; however, in the case of LSC, there is hypergranulosis, which is not a feature of the psoriatic epidermis where a thin stratum granulosum is observed [7,8].

## 3. Psoriasis and Pemphigoid Anti-p200

Anti-p200 pemphigoid is a distinct, rare, blistering disease involving autoantibodies targeting the 200 kDa protein. Due to the immunoreactivity of the γ1 chain of laminin in the serum of most patients, a second name—anti-laminin γ1 pemphigoid—has been proposed. It is more commonly seen in men, and the average age of onset is 65.5 years [9].

Clinically, anti-p200 pemphigoid is characterized by the presence of tense blisters and urticarial eruptions accompanied by pruritus. The involvement of mucous membranes, including the oral cavity, is often present [9].

It is well known that anti-p200 pemphigoid often occurs in patients who have been previously diagnosed with psoriasis, usually within 10 years of the onset of psoriasis, but at the same time, no studies have been conducted to confirm this association. Most of the cases come from Japan, but this may be related to higher detection of cases rather than a true racial predilection. There are several postulated reasons for this coincidence. First is the involvement of metalloproteinases. Metalloproteinase-9 (MMP-9) is involved in the pathogenesis of anti-p200 pemphigoid; at the same time, in patients with psoriasis, the overproduction of many cytokines results in increased expression of MMP-9, and this in turn may stimulate the degradation of laminins, which induces the production of autoantibodies. A second postulated hypothesis is that the phototherapy used in psoriasis treatment may result in a reconfiguration of basement membrane proteins [9].

In the clinical picture of anti-p200 pemphigoid co-occurring with psoriasis, pustules are more frequently observed compared to cases without psoriasis and the lesions are localized primarily on the trunk. Mucosal involvement is rare, and genital involvement is more common. According to literature reports, the most common therapy chosen to achieve remission in such cases has been prednisolone combined with a second immunosuppressive drug or intravenous immunoglobulins or plasmapheresis; however, it is possible to achieve remission with topical therapy alone [9].

## 4. Psoriasis and Pityriasis Rubra Pilaris (PRP)

Pityriasis rubra pilaris (PRP) is an erythematous-squamous dermatosis of uncertain frequency, which occurs in both sexes and in adults as well as in children. It is characterized by the presence of perifollicular hyperkeratotic red-orangish papules that merge together but preserve so-called ‘islands of sparing’, which are areas of non-lesional skin. The classification of PRP according to Griffith initially distinguished five subtypes but later, a sixth was added. The most common type is the classical adult type and in children, the most common is the circumscribed juvenile type [10].

PRP was first described as a type of psoriasis. However, they are now considered separate entities, which some believe may overlap or one transforms into another. There are also some similarities in pathogenesis, as well as in the clinical course and treatment [10].

**Figure 2:**
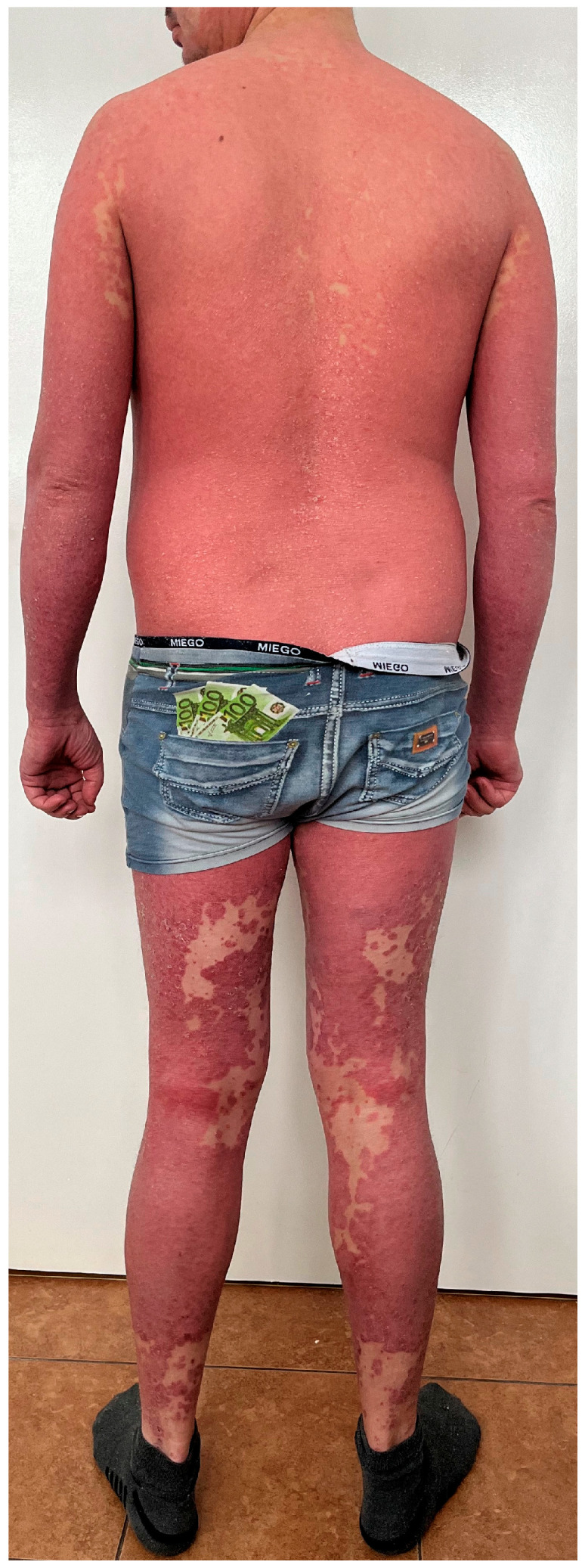
A patient who suffers from psoriasis but due to the presence of several non-lesional skin ‘islands’, PRP was also taken into consideration in the differential diagnosis. Psoriasis was confirmed in a skin biopsy and the patient received risankisumab, which resulted in complete skin lesion resolution.

Psoriasis and PRP share the pathway associated with phospholipases PLA2G2F, PLA2G4D, and PLA2G4E, which are mainly found in the epidermis. Silencing the genes of these three phospholipases results in decreased immune responses and reduced epidermal thickness [11]. Moreover, the triggering of skin lesions by infectious agents and drugs has been observed in both dermatoses [10] but it is more pronounced in psoriasis. As for the clinical picture, both psoriasis and PRP are characterized by the presence of erythematous papules and both can lead to erythroderma [12]. In particular, type I and III PRP can be mistaken for psoriasis. In case of PRP, a distinctive feature is ‘islands of sparing’ and palmoplantar keratoderma. On the other hand, psoriasis is associated with well-defined plaques, thicker silver scales, and sometimes specific nail lesions [10]. Figure 2 shows a patient who suffers from psoriasis but due to the presence of several non-lesional skin ‘islands’, PRP was also taken into consideration in the differential diagnosis.

As for the treatment, the drug of choice for PRP is acitretin, which is also a very popular antipsoriatic drug. If acitretin is not sufficient, methotrexate may be used, which is, again, very frequently prescribed for psoriasis. Phototherapy, which is widely used in psoriasis, is usually indicated in PRP for extensive disease if systemic agents cannot be administered [10]. Similar biological agents can also be used in both conditions; however, in PRP, they are rarely the first-line option and are administered in refractory cases [13] whereas in psoriasis, they could be drugs of choice but it usually depends on the access to biological treatments in particular countries and local guidelines. Reports on PRP treatment most commonly involve infliximab, followed by other anti-TNFα agents, ustekinumab, and the IL-17 inhibitors secukinumab and ixekizumab [13,14]. The clinical trials for PRP treatments are also limited. To date, there have been two completed trials with IL-17 inhibitors: trials for alefacept, which was terminated, and deucravacitinib, which is recruiting [15].

## 5. Psoriasis and Seborrheic Dermatitis

Seborrheic dermatitis is a common skin disease that affects around 1–3% of people across all ethnicities and age groups. The condition develops due to the metabolism of lipids by *Malassezia* sp., which are secreted by sebaceous glands. This process produces free fatty acids, triggering an inflammatory response. The release of pro-inflammatory cytokines stimulates the growth of keratinocytes, disrupting the skin’s protective barrier, resulting in erythema and skin exfoliation. Clinically, seborrheic dermatitis is characterized by red, scaly patches on areas such as the face, scalp, and chest [16]. Treatment typically includes topical anti-inflammatory medications (such as calcineurin inhibitors or corticosteroids) along with either topical or oral antifungal treatments [17].

Sebopsoriasis is a condition that combines two entities—psoriasis and seborrheic dermatitis—and is frequently misleading for physicians and misdiagnosed. Lesions in sebopsoriasis can occur in different areas, which is also suggestive of seborrheic dermatitis, such as scalp, hairline, eyebrows, nasolabial folds, and sternal area [18]. Usually, erythema and oily yellow scales are observed, accompanied by pruritus. Seborrheic dermatitis has been observed to act as a Köbner phenomenon, which is typical for psoriasis and explains the particular clinical presentation. Trichoscopic examination is a very important tool in case of sebopsoriasis. It reveals greasy and silver scales on the surface of erythema with red dots and globules, which are observed at lower magnification, whereas at higher magnification, twisted and bushy red loops can be seen [18]. Microscopic pictures of sebopsoriasis reveal irregular psoriasiform epidermal hyperplasia, spongiosis concentrated around follicular infundibula, parakeratosis overlying follicular ostia, and superficial lymphocytic infiltrations in a focal distribution [19]. Therapy usually consists of topical antifungal agents and topical steroids [18].

## 6. Psoriasis and Inflammatory Linear Verrucous Naevus (ILVEN)

Inflammatory linear verrucous nevus (ILVEN) is a peculiar entity that is nowadays considered a whole heterogeneous group of Blaschko-linear inflammatory conditions [20]. It is a kind of nevus, consisting of red papules and plaques that typically follow the distinctive pattern of Blaschko lines. It is also characterized by pruritus. ILVEN is often present at birth or appears during the first year of life. Over months to years, it spreads along the Blaschko lines before becoming self-limiting in childhood [20]. As for localized disease, surgical excision, dermabrasion, or laser therapy can be used; however, in some cases, systemic treatment options are also applied [21].

ILVEN is also considered to be associated with linear psoriasis. The first hypothesis for this association is that linear psoriasis is an epidermal nevus with psoriasiform features. This form of psoriasis can be associated with just Köbner’s phenomenon, which obviously occurs in this dermatosis; hence, some dermatologists deny the existence of ‘linear’ psoriasis as a separate subtype. However, there have been case reports of psoriatic lesions distributed segmentally [22]. At the same time, others believe that ILVEN is in fact a mosaic form of psoriasis [23]. Another point of view is that they are completely separate entities but can sometimes coexist. Indeed, the clinical presentations, along with the microscopic findings and responses to treatment, are different in psoriasis compared to ILVEN, which suggests that they are distinct [22]. It has been reported that ILVEN can be distinguished from psoriasis through a quantitative immunohistochemical examination. In skin samples of ILVEN lesions, the number of Ki-67-positive nuclei is lower, whereas the number of keratin-10-positive cells and HLA-DR expression are higher compared to samples from psoriatic lesions [24]. In subjects with ILVEN without co-existing psoriasis, the counts of all T lymphocyte subsets and cells expressing NK receptors were decreased compared to psoriasis (excluding CD45RA+ cells). On the other hand, in patients with ILVEN with co-existing psoriasis, the counts of these T lymphocyte subsets had an intermediary position. The most noticeable difference between ILVEN with or without psoriasis is the number of CD8+, CD45RO+, CD2+, CD94, and CD161 cells [24].

The response to ILVEN treatments varies between patients and cases with or without coexisting psoriasis but in general, it is believed to be frequently refractory to therapy [25]. Many agents are commonly used to treat ILVEN and psoriasis, namely topical corticoids, calcipotriol, acitretin, and, more recently, biological drugs [21,25]. Hofer et al. posed the question of whether ILVEN is indeed a distinct type of inflammatory epidermal nevus. They pointed out that some ILVEN cases only partially respond to anti-psoriatic and anti-inflammatory treatments, with minimal improvement in symptoms like pruritus, and such cases are likely linked to an underlying epidermal nevus. On the other hand, when patients show a good response to such treatments, it suggests there may be no underlying nevus, which could create inaccuracies in the dermatological nomenclature. Based on this, Hofer proposed that lesions that respond well to anti-inflammatory drugs should be referred to as inflammatory linear verrucous eruption (ILVE) instead of ILVEN [26].

## 7. Pustular Psoriasis

**Figure 3:**
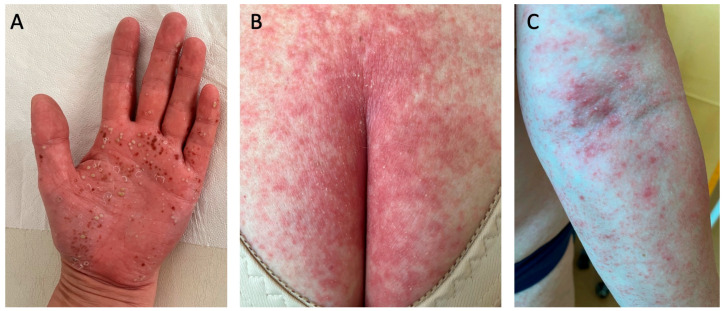
Pustular psoriasis: (**A**) palmoplantar pustulosis palm involvement; (**B**,**C**) von Zumbusch generalized psoriasis. Multiple pustules that had resolved left behind brown scabs, which are typical for palmoplantar pustulosis (**A**). In (**B**,**C**), the patient has suffered from a mild psoriasis for many years, and after experiencing stroke and pulmonary thromboembolism, they developed von Zumbusch generalized psoriasis.

Despite being considered a variant of psoriasis, pustular psoriasis, due to its distinct clinical picture and pathogenesis, is now perceived by many dermatologists as a separate entity. Pustular psoriasis is characterized by the presence of numerous sterile pustules that are either localized or generalized, depending on the subtype [27]. There are two localized variants, namely palmoplantar pustulosis (PPP) (Figure 3A) and acrodermatitis continua of Hallopeau. Another similar term ‘palmoplantar pustular psoriasis’ has been introduced for cases with PPP and concomitant psoriasis, psoriatic arthritis, or a positive family history of psoriasis [28]. The generalized subtype includes the von Zumbusch variant (Figure 3B,C), impetigo herpetiformis, annular pustular psoriasis, and juvenile pustular psoriasis [27]. Plaque psoriasis occurs in approximately 15–83% of patients with generalized pustular psoriasis, whereas psoriatic arthritis occurs in 4–30%. Generalized pustular psoriasis is a chronic disease that manifests with occasional flares that may be potentially life-threatening [29].

Compared to plaque psoriasis, pustular psoriasis is more common among Asians, and the association between smoking and exacerbation of skin lesions (especially in palmoplantar pustulosis) is more pronounced [27]. Moreover, the pathogenesis of these two variants seems different. Psoriasis vulgaris is associated with TNFα, IL-17, and IL-23 [8], whereas PP is associated with IL-36 and IL-1 [27]. Genetic variations in six distinct genes have been associated with pustular psoriasis: genes encoding the interleukin-36 receptor antagonist (*IL36RN*), caspase recruitment domain-containing protein 14 (*CARD14*), adapter protein complex 1 subunit sigma 3 (*AP1S3*), TNFAIP3-interacting protein 1 (*TNIP1*), the serine protease inhibitor SERPINA3, and interleukin-1 receptor antagonist (*IL-1RA* or *IL1RN*) [27]. Nevertheless, efforts have been made to better characterize pustular psoriasis at the molecular level, which would contribute to more in-depth knowledge about the disease and allow for treatment personalization [30]. Several antipsoriatic agents have been widely used in both plaque and pustular psoriasis, namely methotrexate, cyclosporin A, and acitretin; however, there are also drugs that target the cytokines involved in PP. These are spesolimab (IL-36 receptor inhibitor), anakinra (IL-1 receptor antagonist), and canakinumab (IL-1β antagonist) [27]. It has been reported that the response of PP to biological therapy is slower compared to psoriasis vulgaris. Janus kinase (JAK) inhibitors have also been investigated for treating pustular psoriasis and have exhibited beneficial results [28].

## 8. Psoriasis and Sneddon–Wilkinson Disease

Sneddon–Wilkinson disease, also called subcorneal pustular dermatosis, is a rare disease of uncertain frequency that is more common in women and in adults [31]. Sneddon–Wilkinson disease is considered by some to be a member of the family of pustular psoriasis and others believe it is a separate entity [27]. Nevertheless, its clinical presentation resembles that of pustular psoriasis since it involves multiple pustules on an erythematous base, usually located on the flexor surfaces in an annular arrangement [32]. On the other hand, an important differential feature of pustular psoriasis is that the confluent pustules resolve in the form of ‘collarette-like’ exfoliation, which is not typical of Sneddon–Wilkinson disease [33]. In addition, hypopyon pustules occur in Sneddon–Wilkinson disease but not pustular psoriasis [34]. Moreover, subcorneal pustular dermatosis lacks the microscopic features of pustular psoriasis, namely psoriasiform hyperplasia, parakeratosis, and spongiform pustules [31]. Importantly, subcorneal pustular dermatosis is frequently associated with other disorders, especially IgA monoclonal gammapathy, multiple myeloma, and pyoderma gangrenosum, and less often with rheumatoid arthritis or systemic lupus erythematosus [31]. The drug of choice is dapsone [31], which has been successfully used in pustular psoriasis; however, there is discouraging evidence on its use in plaque psoriasis [35]. Phototherapy, such as narrowband UVB and psoralen ultraviolet A (PUVA) therapy as well TNFα inhibitors, can be used in Sneddon–Wilkinson disease and are a common therapeutic option for psoriasis [31]. Recently, abrocitinib, a JAK inhibitor, has been used to successfully treat a case of subcorneal pustular dermatosis [36].

## 9. Psoriasis and Vitiligo

Vitiligo is characterized by the absence of melanocytes, leading to depigmented macules on the skin. It affects both women and men and people of all ethnicities equally. Its pathogenesis involves genetic and autoimmune mechanism, along with modifying external factors. There are two main clinical types, namely non-segmental and segmental vitiligo, as well as a mixed type and unclassified cases. The treatment depends on the type of vitiligo, the surface of the lesions, and their progression. It includes topical anti-inflammatory agents, phototherapy, oral glucocorticoids, and surgical treatment. The newest therapeutic options are JAK inhibitors [37].

A meta-analysis by Yen et al. confirmed the association between psoriasis and vitiligo. Patients with psoriasis had over a two-fold increased risk for developing vitiligo and patients with vitiligo had a greater than 3-fold higher risk of psoriasis [38]. Their bilateral relationship could be attributed to a common genetic background based on a GWAS and shared genetic loci in the major histocompatibility complex [38]. A recent study by Zhang et al. reported that vitiligo and psoriasis share nine differentially expressed genes (DEGs). Only one gene, *AKR1B10,* was upregulated and the rest (*CRABP1, FOXC1, GPM6B, KIT, MLPH, SOX10, TAGLN*, and *TUBB2B*) were downregulated. These overlapping genes are mainly involved in the differentiation of stem cells and melanocytes, as well as in neural crest cell differentiation, development, and migration [39]. Another link is the chronic inflammation observed in both diseases. Both are dependent on the Th1 and Th17 pathways and are associated with T regulatory lymphocytes and increased interferon α secretion [38]. In addition, an autoimmune and autoinflammatory background is suspected to underlie both dermatoses; however, only a few disease-specific auto-antigens have been described so far, and they are not routinely tested in daily clinical practice [8,38,40]. As for clinical similarities, both diseases are characterized by Köbner’s phenomenon [41]. Lastly, some medications are effective in both psoriasis and vitiligo, especially JAK inhibitors as well as phototherapy and topical anti-inflammatory agents [38]. In particular, JAK inhibitors are the most recent advances in the treatment of vitiligo [42]. So far, only one drug from this group has been approved—ruxolitinib—as a topical formula in the form of a cream [43]. Regarding psoriasis, there are case reports on the successful treatment with JAK inhibitors, especially upadacitinib [44]. Upadacitinib has been approved for psoriatic arthritis [45], but not for psoriasis limited to the skin. The only JAK inhibitor approved for psoriasis is so far deucravacitinib.

## 10. Conclusions

**Figure 4:**
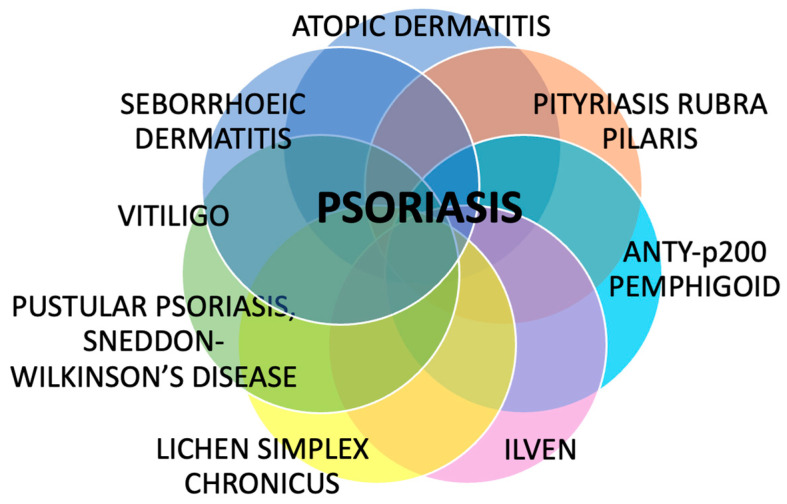
The summary of dermatoses that can coexist with psoriasis. Despite being perceived as solely a skin disease, psoriasis has confirmed or potential associations with many other dermatoses, which have clinical and therapeutic implications.

Despite psoriasis being a common dermatosis, little is known about its associations with other skin diseases. Despite many investigations regarding psoriasis, its potential coexistence and overlap with other skin diseases remain underrecognized and insufficiently explored in the literature. This review summarized several dermatoses that may coexist with psoriasis, overlap with its clinical or microscopic picture, share some pathogenic features, and sometimes lead to diagnostic difficulties and suboptimal treatment (Figure 4).

Understanding the immunological and genetic mechanisms shared by psoriasis and these associated dermatoses—such as Th1/Th17 pathway involvement and IL-17, IL-36, and other shared susceptibility loci—can improve diagnostic accuracy and guide therapeutic decisions. In addition, knowledge of paradoxical reactions during treatment (e.g., induction of eczema or psoriasis and exacerbation of pre-existing lesions) is important in everyday clinical practice; hence, the therapy may sometimes be complicated (e.g., prednisone administration in pemphigoid may exacerbate psoriasis) or, on the contrary, similar drugs could be used to treat two entities (e.g., JAK inhibitors are beneficial for both psoriasis and vitiligo).

Identification of these associations has an academic value as well as significant practical implications. Further research is required to better understand these interrelationships and to develop integrated therapeutic strategies.

## Figures and Tables

**Table 1:** The variants of psoriasis and AD overlap. Based on Tsai et al. [4].

Variant	Clinical Presentation, History, Pathology
1. Psoriasis with features of AD	Nummular psoriasis and psoriatic erythroderma
2. AD with features of psoriasis	More prevalent in Asian populations; more prominent lichenification; more prominent psoriasiform hyperplasia with hypogranulosis, elongation of rete ridge, parakeratosis, and neutrophilic infiltration on pathology
3. Psoriasis dermatitis	Lesions characteristic of psoriasis present at the same time as lesions typical for AD in one patient
4. Eczematoid lesions due to psoriasis therapy	New-onset AD-like lesions, limited or generalized; history of therapy with anti-psoriatic agent, especially anti-TNFα
5. AD therapy-induced psoriasis	New-onset psoriasis-like lesions; history of therapy with dupilumab

## Data Availability

Not applicable.
